# A case of retro‐auricular panfolliculoma mimicking basal cell carcinoma: A rare adnexal tumor with a review of the literature

**DOI:** 10.1002/ccr3.7874

**Published:** 2023-09-07

**Authors:** Saman Al‐Zahawi, Alireza Ghanadan, Ifa Etesami

**Affiliations:** ^1^ Department of dermatology, Razi hospital Tehran University of Medical Sciences (TUMS) Tehran Iran; ^2^ Department of dermatopathology, Razi hospital Tehran University of Medical Sciences (TUMS) Tehran Iran

**Keywords:** basal cell carcinoma, follicular differentiation, immunohistochemistry, panfolliculoma

## Abstract

**Key Clinical Message:**

A rare case of slow‐growing retro‐auricular panfolliculoma is presented. The lesion was biopsied to rule out basal cell carcinoma but histopathology revealed a follicular tumor with differentiation toward all segments of the hair follicle. Panfolliculoma is a benign follicular tumor with no report of recurrence after surgical excision and no malignant transformation of the previously reported cases.

**Abstract:**

Panfolliculoma is a rare slow growing adnexal tumor, characterized by differentiation toward all parts of the hair follicle including the infundibulum, isthmus, stem, and bulbs. Clinically this adnexal tumor may mimick basal cell carcinoma and other adnexal neoplasm including trichoblastoma and trichoepithelioma. In this study, we present a rare case of slow‐growing retro‐auricular panfolliculoma of a 70‐year‐old female mimicking basal cell carcinoma that was successfully excised without recurrence.

## Graphical abstract

Panfolliculoma. (A) Manifesting variable follicular differentiation composed of solid structures of follicular basaloid and squamoid cells (H&E × 10), (B) representing differentiation toward outer root sheath of the hair follicle in level of isthmus (H&E × 20), (C) demonstrating infundibular differentiation composed of cystic infundibulum containing keratin with basaloid cells forming abortive follicular germ cells (H&E × 20), (D) showing more basaloid feature of the tumor (H&E × 10),(E) showing more basaloid feature (H&E × 20).

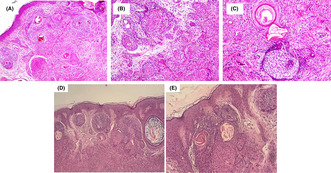

## 1 INTRODUCTION

Panfolliculoma is a very rare, slowly growing, benign neoplasm of follicular differentiation. It is a histological diagnosis, characterized by unique differentiation toward both upper and lower segments of a hair follicle, including the infundibulum, isthmus, stem, and bulbs. This adnexal tumor was described initially by Ackerman et al. in 1993.^1^ it presents clinically as a solitary tumor in the head or trunk.^2^ It is a very rare follicular neoplasia; thus, the objective of this paper is to report a case of slow‐growing panfolliculoma on an ear of an old woman and review previously reported cases in the literature.

## 2 CASE REPORT

**FIGURE 1:**
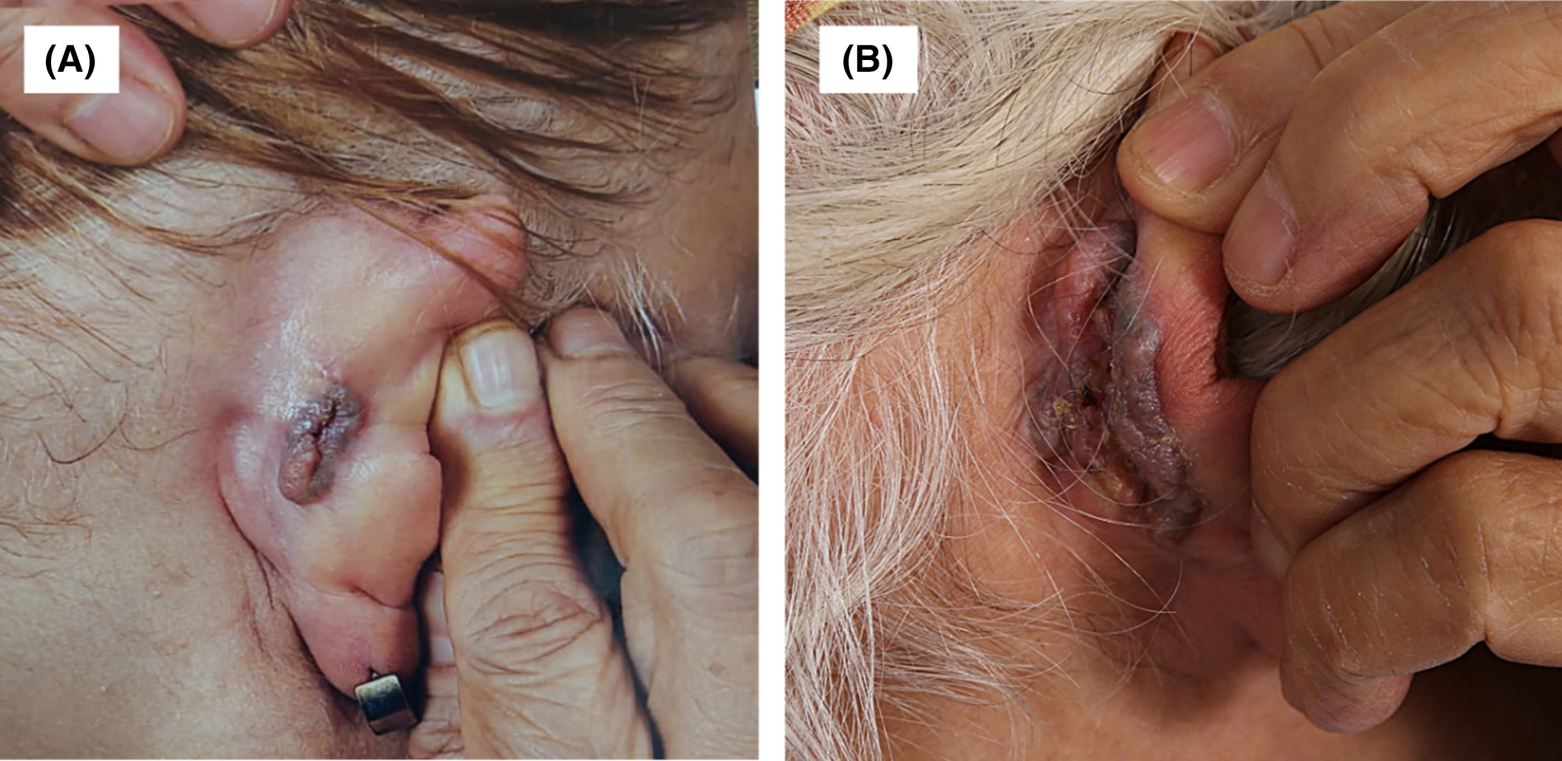
(A) Initial violaceous plaque on the right ear at presentation. (B) Growth of the lesion after 2 years.

A 70‐year‐old female patient visited Razi dermatological hospital in October 2021 for an asymptomatic slow‐growing skin lesion behind her right ear that started more than 3 years ago. She had an initial evaluation in June 2019 in our center and was diagnosed as a case of panfolliculoma with a recommendation to undergo surgical excision, but because of the Covid‐19 pandemic, she postponed her surgical excision. The lesion size grew from nearly 3 × 1.5 cm in June 2019 (Figure 1A) to nearly 6 × 4 cm in October 2021 (Figure 1B), which forced the patient to seek medical attention again. Her second evaluation and biopsy were performed in October 2021, which again was diagnostic of panfolliculoma.

**FIGURE 2:**
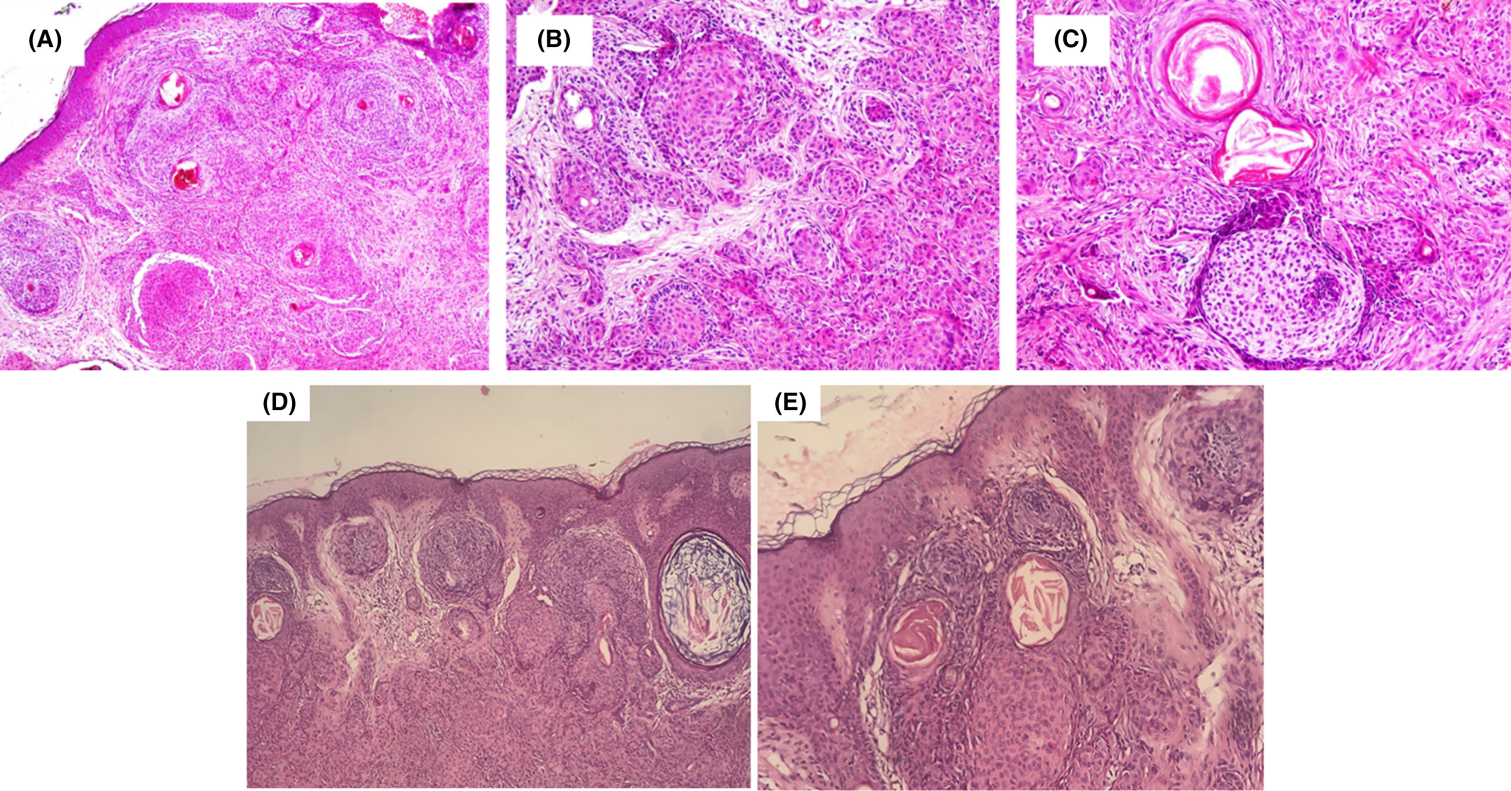
Panfolliculoma (A) manifesting variable follicular differentiation composed of solid structures of follicular basaloid and squamoid cells (H&E × 10), (B) representing differentiation toward the outer root sheath of the hair follicle in the level of the isthmus (H&E × 20), (C) demonstrating infundibular differentiation composed of cystic infundibulum containing keratin with basaloid cells forming abortive follicular germ (H&E × 20), (D) showing more basaloid feature of the tumor (H&E × 10), (E) showing more basaloid feature (H&E × 20).

**FIGURE 3:**
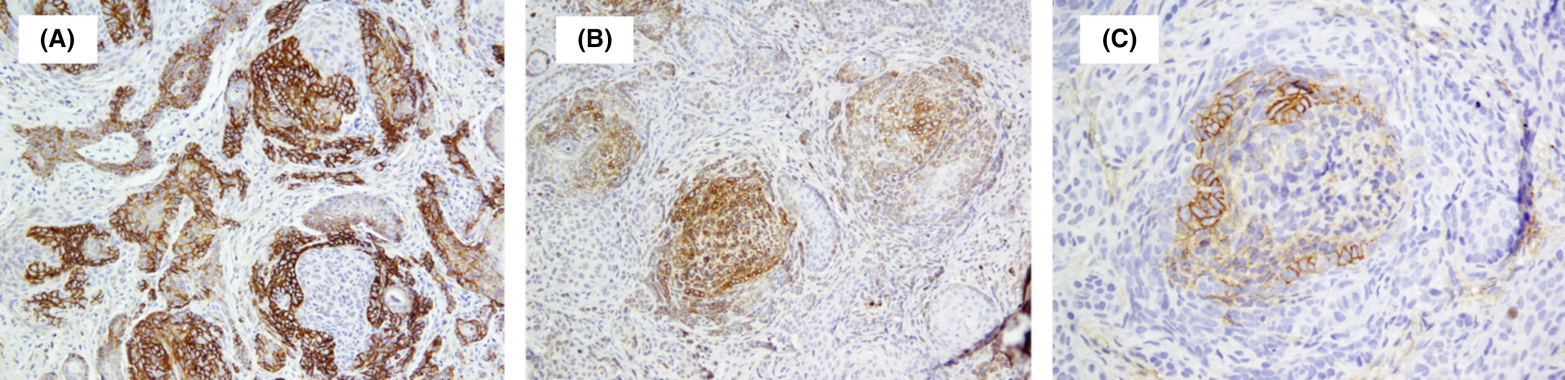
Immunohistochemistry for BerEp4 (A) and Bcl‐2 (B) highlighted basaloid germinative cells and shows focal immunoreactivity for CD‐10 in follicular germ cells (C).

On examination, she had solitary, retro auricular, brown‐violaceous plaque with regular border and depressed center and no signs of bleeding. Biopsy of the lesion revealed a dermal‐based follicular differentiated tumor composed of predominant germinative follicular epithelium with typical follicular stroma and matrical cells (Figure 2A). There were foci of outer root sheath differentiation composed of monomorphic eosinophilic cells with a palisade of nuclei and perifollicular stromal cells (Figure 2B) Also, foci of follicular infundibulum composed of squamous cells with corneocytes and cystic infundibulum containing keratin materials were identified (Figure 2C) and multiple basaloid nests were also identified (Figure 2D,E). Immunohistochemistry study for BerEp4 and Bcl‐2 highlighted follicular germinative cells and CD‐10 demonstrated foci of follicular germ cell and perifollicular stromal cell (Figure 3A–C).

**FIGURE 4:**
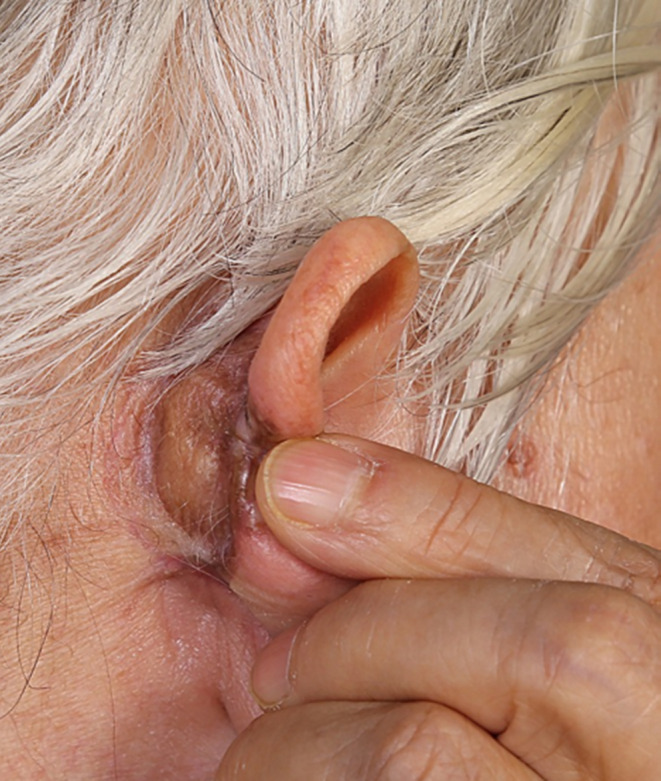
The site of the lesion after excision and skin graft.

After a definite diagnosis of the skin lesion, she was referred to the surgical unit in Razi dermatological hospital and she underwent surgical excision of the lesion with a skin graft from the thigh for correction of the defect (Figure 4).

## 3 DISCUSSION

Panfolliculoma is a very rare, slowly growing, benign neoplasm of follicular differentiation. Panfolliculoma gets its name from its characteristic and unique differentiation toward all parts of both upper and lower segments of a hair follicle, including the infundibulum, isthmus, stem, and bulbs. This adnexal tumor was described initially by Ackerman et al. in 1993. The usual presentation of panfolliculoma is a solitary skin lesion in the head or trunk. In the head, a commonly reported site for panfolliculoma is occiput.^3^, ^4^, ^5^ Although panfolliculoma has been reported in the eyelid, thigh, and forearm,^2^, ^6^, ^7^ per our knowledge, our case is the first case of panfolliculoma to be reported on the ear.

Clinical and histological data of previously reported cases of panfolliculoma are provided in Table 1. Based on the reported cases, the age range of the patients was between 14 and 83 years (with an average of 55 years old), and nearly equal sex distribution (Table. 1).^1^, ^2^, ^3^, ^4^, ^5^, ^6^, ^7^, ^11^

**TABLE 1:** Recent panfollicoma reported cases.

Reference	Age/sex	Clinical presentation	Location	Size	Symptoms	Duration	Type	Treatment	Recurrence
Estrada‐Castanon, R., et al.^2^	58/F	Comedo like	Eye lids	N/A	Mild pruritus	5 years	Cystic	Excision	No recurrence
Hoang et al.^8^	33/F	Cystic lesion	Scalp	3 cm	Asymptomatic	8 months	Cystic	Excision	No recurrence
Harris et al.^6^	81/M	Plaque lesion	Medial thigh	N/A	Asymptomatic	N/A	Intra‐epidermal	Excision	No recurrence
Harris et al.^6^	61/F	SCC like	Lateral thigh	3 mm	Asymptomatic	N/A	Intra‐epidermal	Excision	No recurrence
Juang et al.^9^	14/M	Erythematous nodule	back	4‐6 cm	Discharge and pain	Few months	Cystic	Excision	No recurrence
Preston, et al.^10^	83/M	N/A					Melanocytic	Excision	No recurrence
Fukuyama, et al.^3^	70/M	Dome shaped nodules	Occiput	24 mm	Asymptomatic	10 years	Cystic	Excision	No recurrence
Alkhalidi, et al.^4^	19/F	Scalp swelling	Occiput	8–9 mm	Painful	2 years	Cystic	Excision	No recurrence
Kacerovska, et al.^5^	53/M	N/A	Occiput					Excision	No recurrence
Neill, et al.^7^	64/F	Firm pink papule with slight erosion	Forearm	8 mm	Occasional bleeding and tenderness	11 months	Cystic	Excision	No recurrence
Etesami et al 2022 (our case)	70/F	Plaque like lesion	Ear	4–6 cm	Asymptomatic	3 years	Cystic	Excision	No recurrence

Abbreviation: N/A, not available.

The clinical presentations are variable, it may present as a plaque,^6^ cystic lesion,^4^, ^8^ or nodule,^3^, ^9^ also an SCC‐like lesion,^6^ and comedo‐like lesion^2^ have been reported. The reported size of panfolliculoma lesions varies between 4–6 cm^9^ and 3 mm. The slow‐growing nature of panfolliculoma can be demonstrated in our case in which the initial size was doubled in nearly 2 years. Most recent reported cases of panfolliculoma were asymptomatic^3^, ^6^, ^8^ while pruritus,^2^ discharge and pain,^8^ occasional bleeding, and tenderness^7^ have been reported. Because of the rarity of this adnexal tumor, it is not clear whether a long‐lasting lesion without excision will transform into a malignant lesion or not but the longest reported duration of panfolliculoma at presentation was 10 years without aberrant behavior of the lesion,^3^ while the shortest duration at presentation was 8 months.^8^ Regarding histopathological findings, Shan and Guo classified panfolliculoma into superficial, nodular, and cystic types (12) but recently reported cases have added melanocytic type^10^ and intra‐epidermal type (8) to the histopathological variants. The cystic type was the most common variant reported in the literature followed by the superficial type but Shan and Guo observed that the superficial variant makes up nearly half of the studied cases,^12^ even so, cystic type remains the most common histological variant in the recently reported cases.^1^, ^2^, ^3^, ^4^, ^5^, ^7^, ^8^, ^9^, ^11^

Panfolliculoma is a histological diagnosis but clinically it may look like basal cell carcinoma, other adnexal neoplasms including trichoepithelioma, trichoblastoma, infundibular cyst or even SCC. The histological differential diagnoses of panfolliculoma include trichoepithelioma, trichoblastoma, dilated pore of Winer, epidermal inclusion cyst, pilar cyst, basal cell carcinoma, and trichofolliculoma. Trichoepithelioma and trichoblastoma differ in that they lack more advanced follicular differentiation. Trichoblastoma and trichoepithelioma tend to involve follicular germ differentiation without differentiating toward the infundibulum, isthmus, and outer root sheath. In dilated pore of the Winer there is an infundibular cystic formation filled with keratinous material with no further extension of the cyst to the underlying deep dermis. The cystic form of panfolliculoma may resemble an epidermal inclusion cyst or pilar cyst. Epidermal inclusion cyst may extend to the deep dermis. The cystic structure in epidermal cyst is lined by stratified squamous epithelium containing keratohyaline granules and lamellar‐type keratin with no further follicular differentiation. Panfolliculoma differs from basal cell carcinoma in that it has well‐defined basaloid nests but unlike basal cell carcinoma, the basaloid nests are without clefts and they may contain Merkel cells. Trichofolliculoma consists of a central cystic space at the infundibular level that occasionally contains vellus follicles projecting into the cystic center without deep dermal extension. Panfolliculoma is treated by surgical excision and recurrence of the lesion has not been reported.

## 4 CONCLUSION

In this study, we reported a slow‐growing panfolliculoma on the right ear of an old woman, the lesion size was nearly doubled within 2 years. histologically the lesion showed panfollicular differentiation with positive staining for BerEp4, CD‐10, and Bcl‐2 in IHC. The lesion was excised with no signs of recurrence throughout 12‐month follow‐up period. Based on previous literature, panfolliculoma is a benign tumor, equally occurring in both sexes mainly on the head and neck. Till date no case of recurrence, malignancy, or distant metastasis was associated with this adnexal tumor.

## AUTHOR CONTRIBUTIONS

**Saman Al Zahawi:** Writing – original draft. **Alireza Ghanadan:** Visualization. **Ifa Etesami:** Conceptualization; data curation; formal analysis.

## FUNDING INFORMATION

None.

## CONSENT

Written informed consent was obtained from the patient to publish this report in accordance with the journal's patient consent policy”.

## Data Availability

The authors elect not to share data.
